# Utilization of experimental design in the formulation and optimization of hyaluronic acid–based nanoemulgel loaded with a turmeric–curry leaf oil nanoemulsion for gingivitis

**DOI:** 10.1080/10717544.2023.2184311

**Published:** 2023-02-27

**Authors:** Amal M. Sindi, Khaled M. Hosny, Waleed Y. Rizg, Fahad Y. Sabei, Osama A. Madkhali, Mohammed Ali Bakkari, Eman Alfayez, Hanaa Alkharobi, Samar A Alghamdi, Arwa A. Banjar, Mohammed Majrashi, Mohammed Alissa

**Affiliations:** a Department of Oral Diagnostic Sciences, Faculty of Dentistry, King Abdulaziz University, Jeddah, Saudi Arabia; b Department of Pharmaceutics, Faculty of Pharmacy, King Abdulaziz University, Jeddah, Saudi Arabia; c Department of Pharmaceutics, College of Pharmacy, Jazan University, Jazan, Saudi Arabia; d Department of Oral Biology, Faculty of Dentistry, King Abdulaziz University, Jeddah, Saudi Arabia; e Department of Periodontology, Faculty of Dentistry, King Abdulaziz University, Jeddah, Saudi Arabia; f Department of Pharmacology, College of Medicine, University of Jeddah, Jeddah, Saudi Arabia; g Department of Medical Laboratory Sciences, College of Applied Medical Sciences, Prince Sattam bin Abdulaziz University, Al-Kharj, Saudi Arabia

**Keywords:** Gingivitis, curcumin, emulgels, hyaluronic acid, Box-Behnken design

## Abstract

Numerous problems affect oral health, and intensive research is focused on essential oil–based nanoemulsions that might treat prevent or these problems. Nanoemulsions are delivery systems that enhance the distribution and solubility of lipid medications to targeted locations. Turmeric (Tur)- and curry leaf oil (CrO)–based nanoemulsions (CrO-Tur-self-nanoemulsifying drug delivery systems [SNEDDS]) were developed with the goal of improving oral health and preventing or treating gingivitis. They could be valuable because of their antibacterial and anti-inflammatory capabilities. CrO-Tur-SNEDDS formulations were produced using the response surface Box-Behnken design with different concentrations of CrO (120, 180, and 250 mg), Tur (20, 35, and 50 mg), and Smix 2:1 (400, 500, and 600 mg). The optimized formulation had a bacterial growth inhibition zone of up to 20 mm, droplet size of less than 140 nm, drug-loading efficiency of 93%, and IL-6 serum levels of between 950 ± 10 and 3000 ± 25 U/ml. The optimal formulation, which contained 240 mg of CrO, 42.5 mg of Tur, and 600 mg of Smix 2:1, was created using the acceptable design. Additionally, the best CrO-Tur-SNEDDS formulation was incorporated into a hyaluronic acid gel, and thereafter it had improved *ex-vivo* transbuccal permeability, sustained *in-vitro* release of Tur, and large bacterial growth suppression zones. The optimal formulation loaded into an emulgel had lower levels of IL-6 in the serum than the other formulations evaluated in rats. Therefore, this investigation showed that a CrO-Tur-SNEDDS could provide strong protection against gingivitis caused by microbial infections.

## 1. Introduction

Ever since the World Health Organization broadened the definition of *health* in 1948, there has been an acknowledgement of the quality-of-life factors associated with health (Baiju et al., 2017). As a result, the medical model of health and disease has given way to a biopsychosocial paradigm (Pinheiro et al., 2017). In addition to the absence of disease, oral health also refers to a person’s general health, which allows the person to engage in daily activities such as eating, talking, and smiling, as well as to make creative contributions to society (Nguyen et al., 2017). The health-related quality of life focuses on how illness and the treatment of illness affect the quality of life. Different hypothetical models have been put forth to explain the concept, with Wilson and Cleary’s conceptual model from 1995 being the most thorough (WHOQoL Group, 1995). The definition of the oral health-related quality of life is still nebulous despite extensive study and thousands of articles written about it. In clinical dentistry practice, dental education, and dental research, the patient’s perception of his or her oral health and associated life quality is important (Schwartzmann, 2003). It has been amply demonstrated that oral health issues can affect daily life in a variety of ways. Numerous scales or measurements are available to evaluate this. They vary depending on the type of answer, the number of items, the application setting, and the target population (Gift & Atchinson, 1995).

The most common type of periodontal disease is gingivitis. It can start in early childhood, it peaks in prevalence and severity in early adolescence, and it gradually declines and plateaus until the age of about 20 years is reached (Califano, 2003). The development of a biofilm as a result of inadequate oral hygiene is closely related to the seriousness of the disease. Gingival inflammation can be caused by the biofilm for 10 to 21 days; however, it can be treated by techniques for regulating the biofilm (Van der Velden, 2006). Swollen red gums and hemorrhage are the main indicators of gingivitis. When the teeth are being brushed, the gums may bleed, sometimes for no apparent reason. Because of a lack of pain or other signs, gingivitis frequently goes unnoticed for a long period of time (Blicher et al., 2005).

The green leaves of the curry tree, *Murraya koenigii,* can be eaten as a vegetable and have been shown to have antimicrobial, antinausea, hypoglycemic, antiulcer, antioxidant, apoptotic, and phagocytic properties (Sravani et al., 2015). The chlorophyll in curry leaves has been identified as an antineoplastic agent that also lessens halitosis. Fresh curry leaves can effectively reduce levels of halitosis when chewed for 5 minutes and then rinsed out of the mouth with water, according to experimental tests (Math & Balasubramaniam, 2003). The use of the essential oil from curry leaves for gargling might be beneficial due to its calcium, vitamin C, zinc, and folic acid content. This strengthens the teeth and gums and improves breath freshness. Additionally, its essential oil has antifungal effects and encourages salivation (Nithya et al., 2013).

Turmeric is utilized in medicine as an antioxidant, antibacterial, and anti-inflammatory and for the healing of wounds (Chainani & Wu, 2003). Curcumin, a harmless organic chemical molecule found in turmeric, is responsible for the spice’s significant antibacterial, anti-inflammatory, bactericidal, and antioxidant qualities, among many others. This chemical compound also gives turmeric its vivid yellow color (Toda et al., 1985; Çıkrıkçı et al., 2008). These characteristics made turmeric the ideal component of agents for the management of gingival inflammation (Motterlini et al., 2000). It is a crucial component in mouthwashes, teeth-whitening products, pocket irrigations for removing plaque, and dental sealants for guarding against tooth decay (Ammon et al., 1993). People can lower the risk of cavities and avoid gum disease, gum discomfort, and gum inflammation by consuming turmeric (Chaturvedi, 2009).

Self-nanoemulsified drug delivery systems (SNEDDSs) are transparent colloidal suspensions of oils and aqueous phases stabilized by a surfactant and cosurfactant-based film, encasing droplets smaller than 100 nm (Hosny et al., 2021). Formulations based on nanoemulsions that have specific mucoadhesive qualities can be put into capsules or even introduced into a proper gelling medium to create a nanoemulgel for topical and oral distribution (Date et al., 2010). According to earlier studies, the nanoemulgel had improved permeability and efficiency both *in vitro* and *in vivo*, in addition to the ability to increase the solubility of pharmaceuticals. The nanoemulgel could be readily wiped off whenever necessary and had a longer residence period in the oral mucosa because its appearance and degree of greasiness were not objectionable and its flow behavior was good (Mahmoud et al., 2010). For this reason, many water-in-oil emulsions were frequently utilized in nanoemulgels to effectively deliver hydrophobic medicines. Because of their improved thixotropic and nonstaining qualities, long shelf life, emollient nature, and ease of spreadability, the demand for and application of nanoemulgels has increased significantly (Alexander et al., 2013).

A nonsulfate glycol-amino-glycan called hyaluronic acid (HA) is frequently present in the extracellular matrix of connective tissues. In HA, units of glucuronic acid and N-acetyl glucosamine disaccharide alternate in a linear chain polymer (Goa & Benfield, 1994). Angiogenesis, activation, and migration are encouraged by the improved tissue-healing capabilities of HA, which also allow for stimulation and mild inflammatory responses. The fact that HA is hygroscopic suggests that basal keratinocyte–based proliferation is driving greater re-epithelialization. Since HA regulates the hydration of tissue surrounding an ulcer during inflammation or injury, including it in a formulation to be used in the oral mucosa can be useful (Friedman et al., 2002). Because of their low solubility, commercial oral gels only include medications that have been disseminated into the gel matrix, and this reduces the time that the gel remains in the mouth and necessitates frequent readministration. This issue can be resolved and even improved upon by combining a nanoemulsion with an HA-based gel to create a nanoemulgel, which can be beneficial for the treatment of diseases or for maintaining good dental hygiene (Patel et al., 2011).

The foundation of sound research is the experimental or research design. It plans the data collecting for the experiment, establishes the statistical analysis of the collected data, and directs the interpretation of the findings. It helps readers navigate the ‘Methods’ section when it is adequately presented with in written statement of the experiment, which enhances the efficiency of interaction between writers and readers.

In order to provide a novel formulation for the successful treatment of gingivitis to improve oral health, the current study set out to develop an HA-based nanoemulgel containing curry leaf oil and turmeric in a nanoemulsion (CrO-Tur-NE) and to evaluate its antibacterial, anti-inflammatory, and healing properties.

## 2. Materials and methods

### 2.1. Materials

The source of HA was Merck KGaA (Darmstadt, Germany). Curry oil (CrO) was bought from the Saudi-Indica Natura Company (Jeddah, Saudi Arabia). Ashland delivered Gantrez S-97 (the acid version of methyl vinyl ether and maleic anhydride copolymer) (Tadworth, Surrey, UK). The following substances were bought from Sigma: Turmeric, Tween 80, Span 80, cremophor, isopropyl alcohol, glycerin, and ethanol (St. Louis, MO, USA). Labrasol, Lauroglycol 90, Capryol, Plurol CC49, and Transcutol were graciously provided by Gattefosse (Saint-Priest Cedex, France). The other compounds that were utilized in this experiment were all of analytical grade. Purified water was used in the experiments.

### 2.2. Methodology

#### 2.2.1. Solubility studies

Turmeric (Tur) was tested for solubility in a variety of surfactants, including Tween 80 (hydrophile-lipophile balance value [HLB] = 15), Cremophore EL (HLB = 13), Labrasol (HLB = 12), Lauroglycol 90 (HLB = 3), Capryol (HLB = 5), Span 80 (HLB = 4.3), and Plurol CC49 (HLB = 3). Additionally, cosurfactants such as glycerin, isopropyl alcohol, ethanol, Transcutol, and propylene glycol were used as media for testing Tur solubility. The test involved adding excessive amounts of Tur powder to vials containing 5 ml of each surfactant and cosurfactant. These vials were then securely fastened and kept in an isothermal shaking water bath at a temperature of 25 ± 0.5 °C for 3 days. Following this, specimens were centrifuged at 4000 rpm for 30 minutes, the supernatant was diluted with methanol and filtered through a filter membrane (0.45 µm), the solubility was assessed at λ_max_ at 421 nm using a UV-visible spectrophotometer, and the test was done in triplicate. A 2–24 µg/ml linearity range was discovered, and a correlation coefficient of 0.9989 was obtained. The accuracy was determined to be acceptable (Hosny et al., 2021).

#### 2.2.2. Construction of pseudoternary phase diagrams

The CrO, the surfactant and cosurfactant mixture (Smix), and the deionized water were components of the pseudoternary phase diagrams. Using an aqueous titration method, nanoemulsions containing the CrO, Smix, and deionized water were created. Different ratios of cosurfactant and surfactant were used (i.e. 1:1, 1:2, 1:3, 2:1, 3:1). According to each phase diagram, different weight ratios of CrO and the Smix were combined, and these mixtures were then gradually titrated with the aqueous phase (deionized water) to reach 1 gm total weight of mixture. Then the mixtures were examined for transparency to determine the nanoemulsion area for each tested Smix. This was done with gentle magnetic stirring and without heating the mixtures.

#### 2.2.3. Experimental design and optimization of self-nanoemulsifying formulations

To study the impact of independent variables on the *in-vitro* features and *in-vivo* efficacy parameters for the created SNEDDS formulations, the response surface Box-Behnken design was used with Design-Expert software (version 12.0.6.0; Stat-Ease, Inc., Minneapolis, MN, USA). The amounts of CrO in milligrams (A), Tur in milligrams (B), and Smix 2:1 in milligrams (C) served as the independent variables. The globule size of the manufactured SNEDDS formulations (Y_1_), Tur-loading capacity (Y_2_), interleukin-6 (IL-6) value (Y_3_), and inhibition zone against *Streptococcus mutans* (Y_4_) were the explored dependent responses. Table 1 shows the independent variables and the chosen responses.

**Table 1.** Independent variables and their levels along with dependent variables and their constraints in a Box-Behnken design of nanoemulsion formulations.

Independent variables	Levels		
	−1	0	1
A = Curry leaves oil amount (mg)	120	185	250
B = Turmeric amount (mg)	20	35	50
C = S_mix2:1_ amount (mg)	400	500	600
Dependent variables	Constrains		
Y_1_ = Droplet size (nm)	Minimize		
Y_2_ = Loading Capacity (%)	Maximize		
Y_3_ = Interleukin-6 (U/ml)	Minimize		
Y_4_= Inhibition zone against S. mutans (mm)	Maximize		

#### 2.2.4. Self-nanoemulsifying preparation

The process required creating a loaded nanoemulsion formulation and applying it in two steps (Hosny et al., 2021). The creation of the simple SNEDDS was a component of the initial step. CrO was combined with 400, 500, or 600 mg of the Smix 2:1 in an amount of 120, 185, or 250 mg, respectively, according to the design. In the second step, the simple SNEDDS was combined with the solid ingredient (Tur) in an amount of 20, 35, or 50 mg/g in accordance with the design, as shown in Table 2.

**Table 2.** Box-Behnken design and responses of CrO-Tur–loaded SNEDDS.

Run	A:Curry leaves oil amount (mg)	B:Turmeric amount (mg)	C:S _mix2:1_ amount (mg)	Y_1_:Droplet size (nm)	Y_2_:Loading Capacity (%)	Y_3_:IL-6 (U/ml)	Y_4_:Inhibition zone (mm)	PDI
1	1	0	1	103 ± 2.8	92 ± 0.9	1175 ± 13	17 ± 0.1	0.17
2	0	0	0	96 ± 1.4	84 ± 1.1	1470 ± 11	12 ± 0.7	0.15
3	−1	1	0	74 ± 0.8	55 ± 1.5	1400 ± 5	14 ± 0.3	0.22
4	1	−1	0	118 ± 5.1	90 ± 2.0	1390 ± 4	15 ± 0.2	0.19
5	0	0	0	97 ± 1.8	85 ± 1.4	1500 ± 10	13 ± 0.5	0.26
6	0	1	1	85 ± 0.5	60 ± 0.7	1000 ± 4	19.5 ± 0.5	0.33
7	−1	0	−1	89 ± 1.2	82 ± 0.9	2400 ± 20	8 ± 0.4	0.18
8	0	0	0	95 ± 3.3	84 ± 1.3	1412 ± 15	13.5 ± 0.6	0.20
9	−1	−1	0	72 ± 0.19	90 ± 2.1	3000 ± 25	6 ± 1.2	0.27
10	−1	0	1	65 ± 2.2	81 ± 1.5	2350 ± 18	9 ± 0.9	0.35
11	0	−1	1	84 ± 1.3	85 ± 0.6	1980 ± 20	10 ± 0.5	0.19
12	0	1	−1	109 ± 1.9	60 ± 1.9	1011 ± 8	19 ± 0.7	0.24
13	1	0	−1	136 ± 2.2	93 ± 2.1	1230 ± 12	16 ± 0.4	0.15
14	0	−1	−1	105 ± 2.8	88 ± 1.8	1900 ± 17	11 ± 0.6	0.29
15	0	0	0	97 ± 3.0	86 ± 1.4	1400 ± 9	12.5 ± 0.3	0.31
16	1	1	0	122 ± 1.5	80 ± 1.3	950 ± 10	20 ± 0.5	0.23

#### 2.2.5. Characterization of the fabricated Tur-CrO-SNEDDS formulations

##### 2.2.5.1. Droplet size assessment (Y_1_)

By combining 100 µl of the formulation with 900 µl of purified water in a volumetric flask, the droplet size of the CrO-Tur-SNEDDS formulations was determined. A Microtrac Zetatrack particle size analyzer used 100 µl of the dispersed sample after the formulation had undergone vigorous mixing to estimate the globule size and polydispersity index (PDI) (Microtrac, Inc., Montgomeryville, PA, USA) (Salem et al., 2022).

##### 2.2.5.2. Evaluation of the CrO-Tur-SNEDDS formulations’ drug-loading capacity (Y_2_)

The amount of Tur loaded in each SNEDDS mixture was calculated by independently dissolving a predetermined quantity of Tur in a plain SNEDDS in accordance with the experimental design. The mixtures were put into vials and kept in a shaking water bath at 25 ± 2 °C for 24 hours. The prepared mixtures were centrifuged for approximately 15 minutes at 4500 rpm after reaching equilibrium. The precipitates were gathered, thoroughly washed, and then dissolved in methanol. The medication was extracted, and the amount of Tur was quantified via an aforementioned high-performance liquid chromatography (HPLC) technique (Mudge et al., 2016). In this technique, the separation was accomplished on a C18 column (Phenomenex, Torrance, CA, USA). The mobile phase consisted of (A) 0.1% formic acid in water and (B) 0.1% formic acid in acetonitrile, applying a flow rate of 1.4 ml/min (0 to 4.1 min). To boost the sample throughput, the flow rate was increased to 1.75 ml/min with an injection volume of 0.8 µl and a column temperature of 55 °C. At 425 nm, the UV-Vis detection was observed. The formula below was used to calculate the drug-loading capacity (Sun et al., 2012):

(1) Drug−loadingcapacity = Drug content in NE formulation (mg)Total formulation wt (mg)×100

##### 2.2.5.3. IL-6 level evaluation (Y_3_)

###### 2.2.5.3.1. Animal handling and care. 

The treatment and care of the animals was done in accordance with the regulations set forth by the Cairo Agriculture for Experimental Animals’ Animal Ethics Committee, Cairo, Egypt, Approval No (113-10-22). According to the Declaration of Helsinki and its Guiding Principles for the Care and Use of Animals (NIH Publication No. 85-23, 1985 revision), researchers adhered to certain rules. The experimental rats were housed in cages in the laboratory with free access to food and water. In order to reduce suffering, proper care was given to the animals. Prior to the experiment, the animals were acclimated for at least 14 days under typical circumstances of 25 °C temperature and 55.5% relative humidity with a 12-hour cycle of light and darkness. The experiment involved 48 adult rats, which were divided into 16 groups of 3 rats each. Every group received treatment using a formulation chosen based on the study design. The following steps were taken to construct the *in-vivo* periodontitis model. Chloral hydrate 10% was injected intraperitoneally to anesthetize the rats. After that, they were secured in the supine posture, and a homemade mouth expander was used to open the mouth. A gingival separator was used to separate the gums and totally expose the bilateral maxillary first molars. Each rat in the group had both of their maxillary first teeth tied together for a period of 2 weeks using an orthodontic steel wire (0.2 mm in diameter). By placing the ligation wires in the gingival sulcus, the ligation did not interfere with the rats’ ability to feed or harm their buccal mucosa. In other groups, the periodontitis model was developed in a single day. The buccal, lingual, and mesial gingiva of the maxillary molars, as well as the adjacent tooth space between the maxillary molars, were then injected with variously produced CrO-Tur-SNEDDS (60 µl for each tooth).

###### 2.2.5.3.2. Il-6 level determination. 

The quantitative sandwich enzyme immunoassay technique is used in IL-6 determination (R&D Systems, Inc., Minneapolis, MN, USA). A microplate had been pre-coated with a monoclonal antibody that was specific to rat IL-6, which was linked to any rat IL-6 present in the sample. An enzyme-linked polyclonal antibody specific for rat IL-6 was added after any unbound substances were removed; the blue product changed to yellow, and the intensity of the color was assessed to ascertain the amount of IL-6 in each of the test samples (Hosny et al., 2020).

##### 2.2.5.4. Antibacterial activity evaluation (Y_4_)

The disk perfusion method was used to assess the CrO-Tur-SNEDDS formulations’ antibacterial efficiency. As test bacteria, *Streptococcus mutans*, which is frequently detected in cases of gingivitis, was used. The Clinical and Laboratory Standard Institute (CLST) suggested that the *S. mutans* suspension be prepared so that the turbidity was equal to the 0.5 McFarland turbidity standards before being plated onto Muller-Hinton agar. The disk specimen was positioned in the center of the agar plate and incubated at 37 ± 0.5 °C for 24 hours. The disk specimen had a diameter of 10 mm. A clean (inhibition) zone free of colonies could be visible all around the disk specimens if inhibitory concentrations were attained. For each CrO-Tur-SNEDDS that was put to the test, the breadth of the inhibition zone was measured (Hosny et al., 2021).

#### 2.2.6. Optimization of the CrO-Tur-SNEDDS

For the analysis of variance (ANOVA) of the resulting models, parameters including the *F*-ratio, *p*-value, and degrees of freedom were computed for all independent variables and their interactions. The responses were utilized to choose the model that best fit the obtained data based on the results. *P*-values below .05 specifically showed that the investigated model terms were significant. The model fitness was further evaluated using the coefficient of variation percentage (CV%) values, determination coefficient values, predicted R-squared value, and adjusted R-squared value. Based on the fact that the generated CrO-Tur-SNEDDS formulations could accomplish the four objectives for the dependent variables—that is, minimize the globule size and IL-6 result values and maximize the drug-loading capacity and the inhibitory zone against *S. mutans*—the best variables were chosen.

#### 2.2.7. Characterization of the optimized CrO-Tur-SNEDDS

The produced and evaluated optimized formulation’s globule size, drug-loading capability, IL-6 estimate, and antibacterial efficacy against *S. mutans* were all measured and compared with the theoritical values of the same responses suggested by the software. The formulation was next tested for centrifugation stability and thermodynamic stability to see how well it would become integrated into the HA gel base.

##### 2.2.7.1. Freeze-thaw cycle

For the developed ideal nanoemulsion formulation, three freeze-thaw cycles between −25 and +25 °C, with storage at each temperature for 48 hours, were determined. Particle size analysis was used to measure the globule size each time. The polydispersity index (PDI) assay was used to gauge the degree of uniformity in a nanoemulsion’s globule size. A lower polydispersity score denoted a higher degree of consistency in the nanoemulsion’s globule size (Hosny et al., 2021).

(2) Stability index = ([Initial size−Change in size]/Initial size)×100

##### 2.2.7.2. Centrifugation

The best developed CrO-Tur-SNEDDS formulation was centrifuged at 3500 rpm for 20 minutes in order to check for any indications of instability, such as cracking, creaming, or phase separation.

#### 2.2.8. Preparation of the hyaluronic acid nanoemulgel

An aqueous solution was made using 1.5% HA and 0.5% Gantrez S-97 (Mw = 1.2 106 Da) as cross-linking agents. The mixture was then put into casted molds that were 10 × 10 in size and left to dry for roughly 48 hours. The dry films were cut into 1-cm squares and heated to 81 °C for 24 hours. The hydrogels were made using a microwave-assisted method. The films were placed in an oven, and utilizing the oven’s maximum output power, the cross-linking of the films took 1 hour. In order to create the polymeric solution, the pH-induced hydrogel (HA) was mixed with 18 ml of phosphate buffered saline (PBS), which has a pH of 4.5, at a temperature of 36 ± 2 °C. The solution was allowed to hydrate overnight while being agitated at 50 rpm. About 12 ml of the optimized NE was added to the polymeric solution in order to load the optimal CrO-Tur-SNEDDS into the HA hydrogel base (NG1). After being put into the hydrogel, the size of the CrO-Tur-SNEDDS was assessed to ensure that no changes had occurred. For further testing, the final CrO-Tur-SNEDDS–loaded HA hydrogel was kept in the refrigerator. Three more gel formulations (NG2 to 4) were created for the purpose of comparison. In NG2, an HA gel was created utilizing the Tur in powder form and CrO in oil form rather than the SNEDDS. For NG3, hydroxypropyl cellulose (HPC) was used in place of HA to generate the nanoemulgel that was loaded with the optimized CrO-Tur-SNEDDS. In contrast, NG4 was produced using oleic acid rather than CrO as a component of the SNEDDS and loaded in an HA gel base. The produced gels were then evaluated for the following characteristics.

##### 2.2.8.1. In-vitro release of Tur from the optimized nanoemulgel formulation

In this study, the USP Dissolution Tester (Apparatus I) was used. Instead of using baskets, glass cylindrical tubes measuring 2.7 cm in diameter and 10 cm in length were attached to the spinning shafts and tightly covered with semipermeable membranes (100-m pore size). These tubes contained the tested compositions. Ten grams of the examined formulations (containing 375 mg of Tur) were evaluated against a dispersion of 375 mg of Tur in 10 ml of distilled water. The tubes were then submerged in 50 ml of PBS (pH 6.8). The medium was agitated at a speed of 25 rpm while the release investigation was conducted at 37 ± 0.5 °C (Alhakamy et al., 2022). For 1 hour, samples were taken out of the dissolving milieu at various intervals. Using the previously mentioned HPLC technique, the absorbance was measured to determine how much Tur was released.

##### 2.2.8.2. Ex-vivo transmucosal permeation study

*Ex-vivo* tests were performed on the Tur aqueous suspension and various manufactured formulations (Xiang et al., 2002), each of which included 37.5 mg/ml of Tur. An automatic Franz diffusing cell (MicroettePlus, Paso Robles, CA, USA) was used as the apparatus, and sheep buccal mucosa from a nearby slaughterhouse was used as the model permeation membrane. The Franz diffusion cell’s donor and receptor chambers were properly positioned to encompass the processed sheep buccal mucosa (2 × 2 cm) (1.75 cm^2^). The medium was agitated at a rate of 400 to 450 rpm while being kept at a temperature of 37 ± 0.5 °C in the receptor chamber, which contained 8 ml of PBS (pH 6.8). At regular intervals, finite aliquots were automatically taken, and the aforementioned HPLC technique was used to quantify the Tur content. Plotting the cumulative amount of permeated Tur (Q_24_) per unit of area against time let researchers better understand how the drug was dispersed across the mounted mucosa. From the acquired diffusion data, significant parameters such as the Jss (steady-state flow), Pc (permeability coefficient), EF (enhancement factor), and D (diffusion coefficient) were determined. Plots were made of the comparative permeation patterns for various formulations. The following equation as used to determine the percentage of permeated Tur and the overall amount of Tur distributed across the receptor chamber (Hosny et al., 2021):

(3) $$\mathrm{Percentage}\;\mathrm{permeated}\frac{Tp}{Ti}\times 100$$

where Ti was the initial quantity of Tur in the donor compartment and Tp was the amount of Tur that permeated the receptor compartment.

##### 2.2.8.3. Determination of the zone of inhibition against S. mutans

In addition to the aqueous dispersion of Tur, the antibacterial activity of the gel formulations was assessed by the disk diffusion method, as previously stated.

##### 2.2.8.4. IL-6 serum level determination

This experiment involved the test described in Section 2.2.5.3 conducted with a fresh bunch of 6 groups of rats and each group had 3rats. According to the outcomes of the first stage of the animal test performed during the characterization of the SNEDDS formulations, the first group was given the software’s recommended formulation, which was incorporated into an HA gel base. The second group was treated using the software’s recommended formulation, which was loaded into a base of HPC gel. The third group received a topical application of HA gel filled with the best formulation made without Tur. The fourth group received a topical application of HA gel containing the optimal formulation fabricated with oleic acid rather than CrO. The fifth group was treated with an aqueous dispersion of Tur. The sixth group was treated with topical normal saline.

## 3. Results and discussion

### 3.1. Solubility study of Tur in different surfactants and cosurfactants

The findings showed that Tur was more soluble in surfactants with a low HLB than in those with a high HLB. However, since the HLB for a surfactant must be 10 or above to generate an oil/water (o/w) nanoemulsion (Azeem et al., 2009), a mixture of two surfactants was prepared and blended in the right proportions to create a surfactant mixture with an HLB of 10. So, a surfactant blend was developed by admixing Plurol and Labrasol in a ratio of 1:4.5 to prepare a mixture with an HLB value of 10, and the solubility of Tur in this new blend was found to be 270 mg/ml.

**Figure 1.**
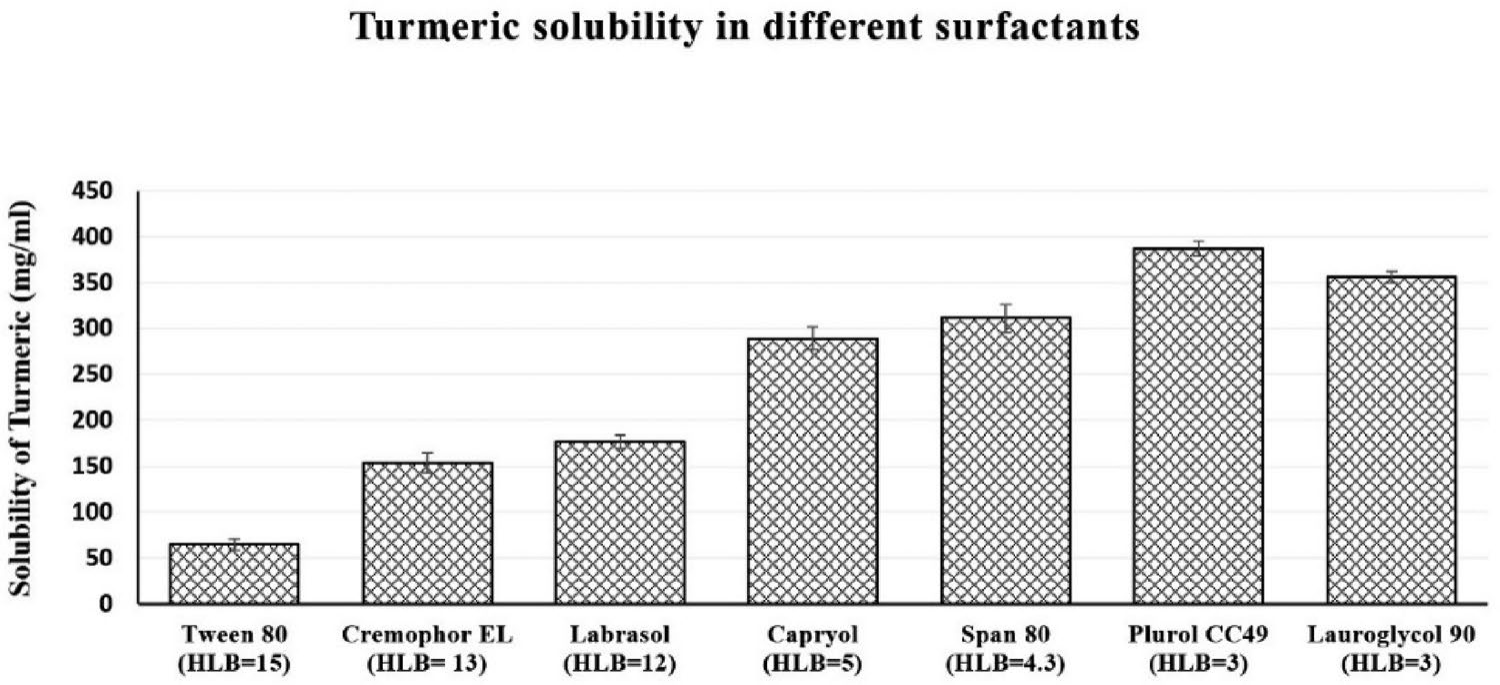
Solubility of Tur in different surfactants.

**Figure 2.**
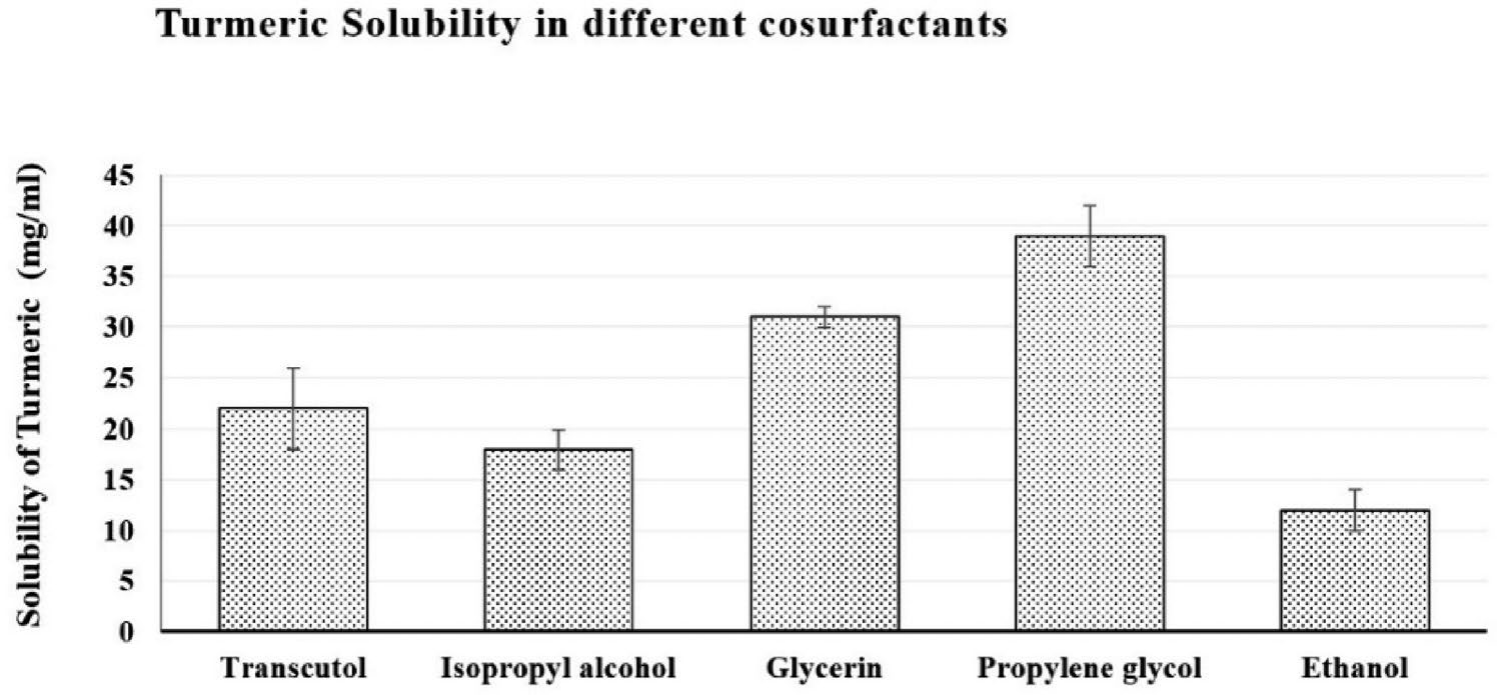
Solubility of Tur in different cosurfactants.

To evaluate the Tur solubility in cosurfactants, propylene glycol was chosen according to the results of the solubility study. Figures 1 and 2 reveal the outcomes of the Tur solubility tests in surfactants and cosurfactants, respectively.

### 3.2. Construction of the pseudoternary phase diagrams

**Figure 3.**
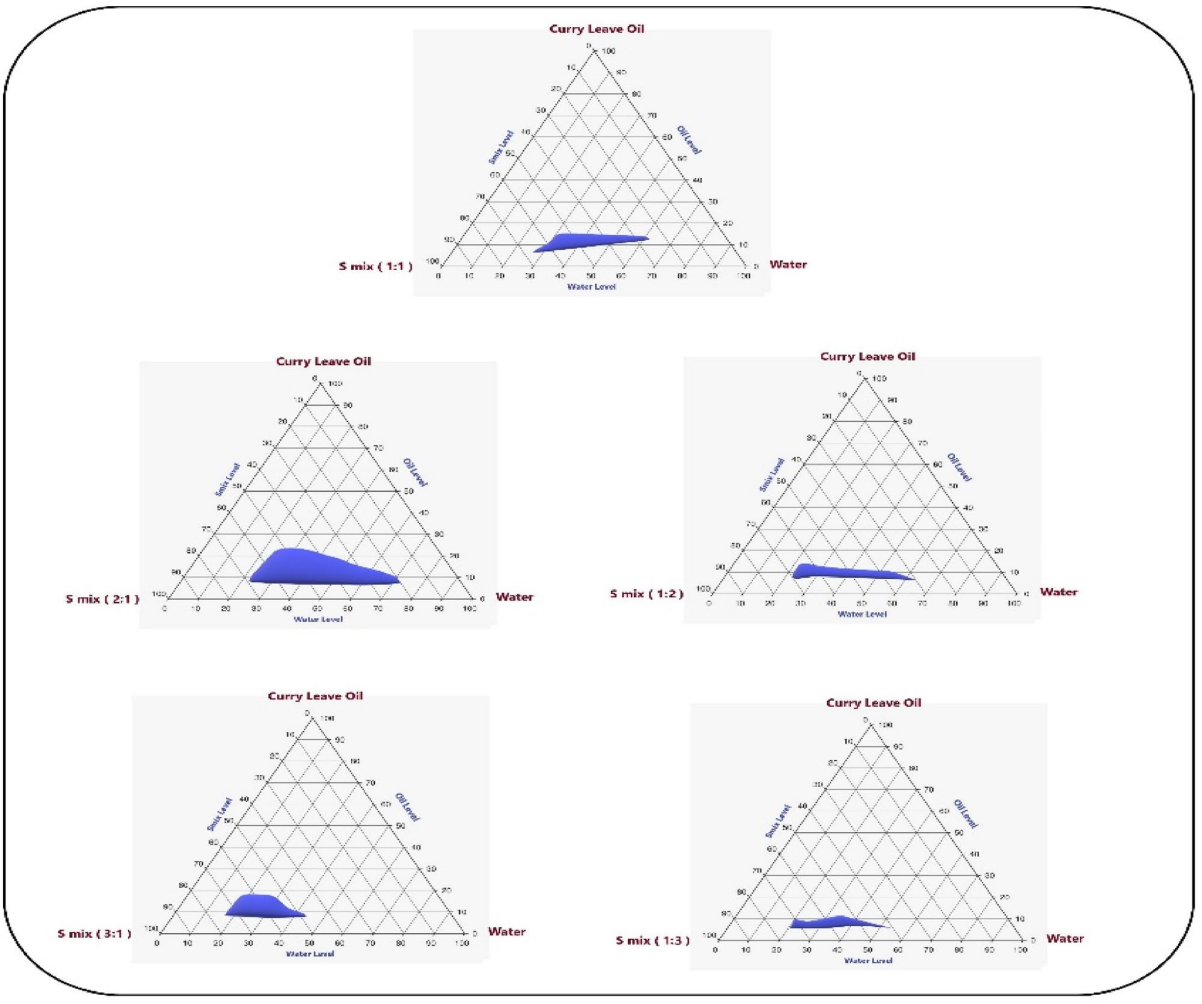
The pseudoternary phase plot of CrO, the Smix, and deionized water.

When Smix 1:1 was replaced with Smix 2:1 or Smix 3:1, the nanoemulsion region grew longer and wider; Smix 2:1, however, was associated with a greater nanoemulsion region and an oil content that could reach 25% in the nanoemulsion area. Switching from Smix 1:1 to Smix 1:2 or Smix 1:3, which contained more cosurfactant than surfactant, caused shrinkage in the nanoemulsion region and restricted the amount of oil that could be loaded into the nanoemulsion to 10%. Figure 3 shows the pseudoternary phase diagrams.

### 3.3. Characterization of the fabricated CrO-Tur-SNEDDS

#### 3.3.1. Globule size determination (Y_1_)

The resulting nanoemulsion’s PDI values ranged from 0.12 to 0.35, and its droplet sizes ranged from 72 ± 0.19 nm to 136 ± 2.2 nm (Table 2), with PDIs of between 0.12 and 0.35 demonstrating the formulations’ acceptable homogeneity, stability, and size distribution.

The obtained globule size information was applied to a linear polynomial analysis model. According to the chosen mathematical design, the researched model was successful in determining the significant influence of the amounts of CrO (A), Tur (B), and Smix 2:1 (C) on the CrO-Tur-SNEDDS formulations’ droplet sizes. The selected model had an adjusted R-squared value of 0.9841 and a predicted R-squared value of 0.9737, which were highly linked, as shown in Table 3. The equation below was created using an ANOVA data analysis:

(4) Droplet size= +96.69+22.38 A+1.38 B−12.75 C

**Table 3.** Regression analysis results for the Y_1_, Y_2_, Y_3_, and Y_4_ responses.

Dependent variables	R^2^	Adjusted R^2^	Predicted R^2^	F-value	p-value	Adequate precision
Y_1_	0.9873	0.9841	0.9737	309.85	0.0001	58.7257
Y_2_	0.9943	0.9858	0.9276	76.78	0.0001	34.9945
Y_3_	0.9952	0.9879	0.9417	27.23	0.0007	41.2653
Y_4_	0.9818	0.9546	0.7784	43.94	0.0001	22.0029

**Figure 4.**
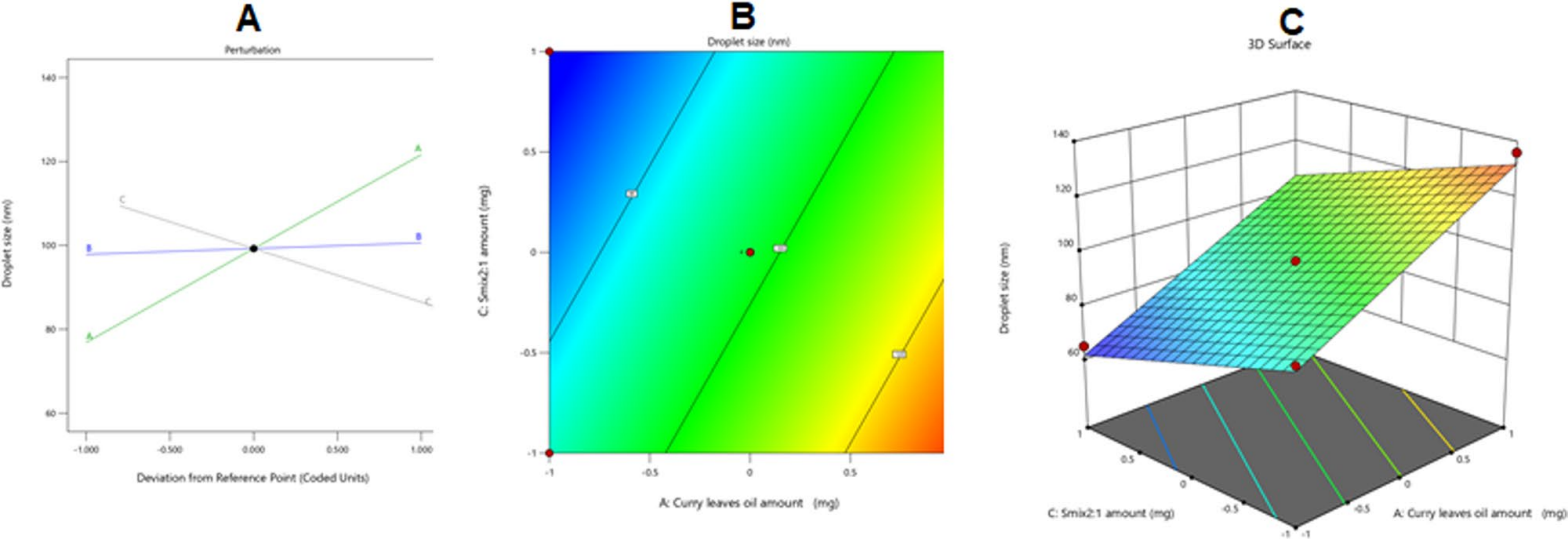
Perturbation (A), contour (B), and 3D surface (C) plots showing the effects of several independent parameters on the size of the droplets in various CrO-Tur-SNEDDS formulations.

The size of the CrO-Tur-SNEDDS droplets was affected by the parameters, as seen in the perturbation, three-dimensional (3D) surface, and contour plots in Figure 4. These graphs show how the formulations’ CrO and Smix 1:2 content affected the globule size of the nanoemulsions that were produced.

The aforementioned equation and graph show that the CrO and Smix 1:2 (i.e. factors A and C, respectively) significantly affected the size of the droplets (*p*-value < .0001). Figure 4A illustrates how the increase in surfactant limits reduced the size of the globules by lowering the interfacial tension between the aqueous and nonaqueous phases, which in turn led to smaller droplets (Rizg et al., 2021). The rise in droplet size in connection with an increased CrO amount may be explained by the fact that increasing the percentage of oil lowered the levels of the used Smix and, as a result, inhibited its ability to minimize the droplet size. This resulted in the production of larger oil globules, a result similar to the results described in the literature (Pavoni et al., 2020). Increasing the oil content might have created larger droplets by providing the medication with more room in which to be fitted. Additionally, raising the Tur amount (i.e. factor B) may have caused the droplets to expand and the emulsion droplets to have a larger diameter; however, this effect was found to be minor (*p*-value = .1300). In the literature, similar outcomes were found (Hosny et al., 2020; Rizg et al., 2021).

#### 3.3.2. Evaluation of the CrO-Tur-SNEDDS formulations’ drug-loading capacity (Y_2_)

The amount of Tur loaded into the developed nanoemulsion formulations was found to range between 55 ± 1.5 and 93 ± 2.1%, as shown in Table 2. The resulting drug-loading data were analyzed using a polynomial quartic model. The principal effects of the CrO (A), Tur (B), and Smix 2:1 (C) levels were examined using the model to valuate the CrO-Tur-SNEDDS drug-loading capacity. The chosen model, shown in Table 3, produced an adjusted R-squared value of 0.9858 and a predicted R-squared value of 0.9276. Data collected were analyzed using ANOVA, which elaborated the equation shown below:

(5) $$\mathrm{Loading}\;\mathrm{capaccity}\;=\;+84.76+5.88\;A-12.25\;B-0.6250\;C+6.26\;AB+0.0000\;AC+0.7500\;BC+3.88\;A^{2}\;–\;9.88\;B^{2}\;–\;1.62\;C^{2}$$

**Figure 5.**
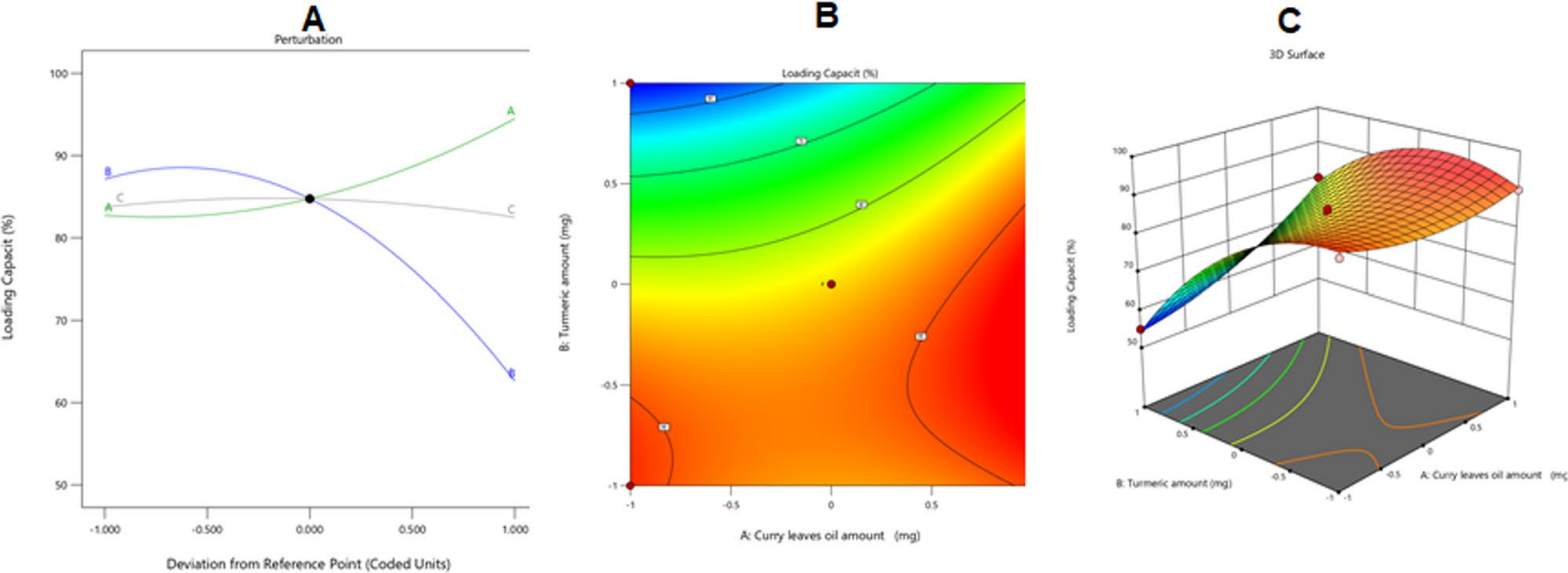
Perturbation (A), contour (B), and 3D surface (C) plots showing the effects of several independent parameters on the Tur-loading capacity in various CrO-Tur-SNEDDS formulations.

Figure 5 shows the perturbation, contour, and 3D surface graphs that reveal how the examined parameters affected the Tur-loading capacity in the developed NEs.

As seen in the previous plot, factor A (CrO level) dramatically increased the amount of Tur entrapped in the nanoemulsion’s droplets (*p*-value < .0001). This could be attributed to the lipophilic properties of the Tur, which increased the oil’s ability to encapsulate the desired medication. Additionally, increasing the oil content in the emulsion would reduce the quantities of the Smix employed in conjunction with it, reducing its ability to cause leakage of the medication into the nearby aquatic environment (Gao et al., 2019).

#### 3.3.3. IL-6 Level evaluation (Y_3_)

Cells frequently produce IL-6, a molecule that aids in controlling immune responses. A helpful marker of inflammation and immune system activation, IL-6 is typically raised in response to microbial invasion, inflammation, immune system disorders, and, sometimes, malignancy (Tanaka et al., 2014).

Il-6 serum levels in animals treated with the developed formulations oscillated between 950 ± 10 and 3000 ± 25 U/ml. Based on the chosen statistical design and the measured mean IL-6 serum levels, a quadratic model of polynomial equations was developed to assess the influence of the investigated independent factors on IL-6 serum levels. The model’s adjusted R-squared value of 0.9879 was extremely near the expected R-squared value of 0.9417 (Table 3). The following equation was produced by an ANOVA analysis of the collected data.

(6) $$Il-6\;\mathrm{level}\;=\;+1445.50-550\;A-488.63\;B-4.50\;C+290.00\;AB-1.25\;AC-22.75\;BC+2277.75{\;A}^{2}-38.25B^{2}+65.50\;C^{2}\;$$

**Figure 6.**
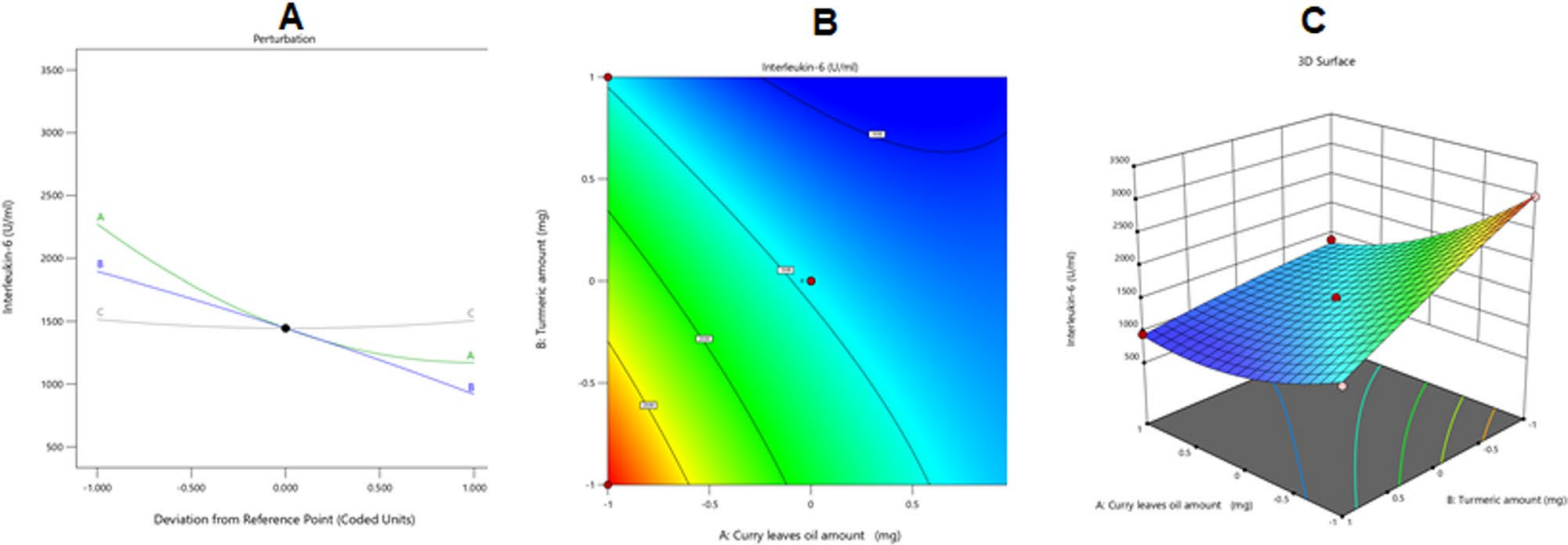
Perturbation (A), contour (B), and 3D surface (C) plots showing the effects of several independent parameters on the Il-6 serum levels in animals receiving various CrO-Tur-SNEDDS formulations.

Figure 6 shows the impact of the investigated independent variables on the IL-6 serum levels as revealed by the perturbation, contour, and 3D surface graphs.

As might be perceived from the preceding curve and equation, factor A (CrO amount) and factor B (Tur amount) exerted a significantly negative impact on IL-6 serum levels in tested rats, and this signified their anti-inflammatory impacts. The antiinflammatory effect of Tur might be associated with the effect of the curcumin it contained, which has been reported to result in good anti-inflammatory effects.

By controlling inflammatory signaling pathways and reducing the generation of inflammatory mediators, curcumin exerts anti-inflammatory actions (Medzhitov, 2008). Activator protein 1 (AP-1) and other signaling pathways are controlled by curcumin’s binding to Toll-like receptors (TLRs), and this also controls nuclear factor kappa-B (NF-B), mitogen-activated protein kinases (MAPKs), and other signaling pathways (Rahimifard et al., 2017; Gao et al., 2019; Zhang et al., 2019) that control inflammatory mediators and treat inflammatory diseases. Additionally, by acting on the PPAR-gamma (peroxisome proliferator–activated receptor gamma) receptor, curcumin can inhibit NF-B (Li et al., 2019; Zhu et al., 2019). By controlling the Janus kinase/signal transducer and activator of transcription (JAK/STAT) inflammatory signaling pathway, curcumin can have anti-inflammatory effects (Kahkhaie et al., 2019; Ashrafizadeh et al., 2020). Factor A (CrO amount) was shown to have anti-inflammatory actions as proven by the IL-6 seerum level test. This might be due to its terpene alkaloids, which might have a role in suppressing the synthesis and activity of prostaglandins, proteases, or lysosomes (Gupta et al., 2010). Factor C (Smix 2:1 amount) was found to have a nonsignificant action on IL-6 serum levels in tested rats (*p*-value = .8483).

#### 3.3.4. Antibacterial activity evaluation (Y_4_)

The inhibition zones against *S. mutans* produced by the nanoemulsionE formulations were found to fluctuate from 6 ± 1.2 to 20 ± 0.5 cm, as shown in Table 2.

The collected zone inhibition data were fitted to a unique quartic polynomial model. The chosen statistical model was used to analyze the CrO-Tur-SNEDDS formulations’ growth zone inhibition capabilities in order to evaluate the main effects of the amount of CrO (A), Tur (B), and Smix 2:1 (C). The chosen model had an adjusted R-squared value of 0.9546 and a predicted R-squared value of 0.7784, as shown in Table 3. Data collected were analyzed using ANOVA, which elaborated the equation shown below:

(7) $$\mathrm{Inhibition}\;\mathrm{zone}=\;+12.75+3.88\;A+3.81\;B+0.1875\;C-0.7500\;AB+0.0000\;AC+0.3750\;BC-0.6875{\;A}^{2\;}+1.69\;B^{2}+0.4375\;C^{2}\;$$

**Figure 7.**
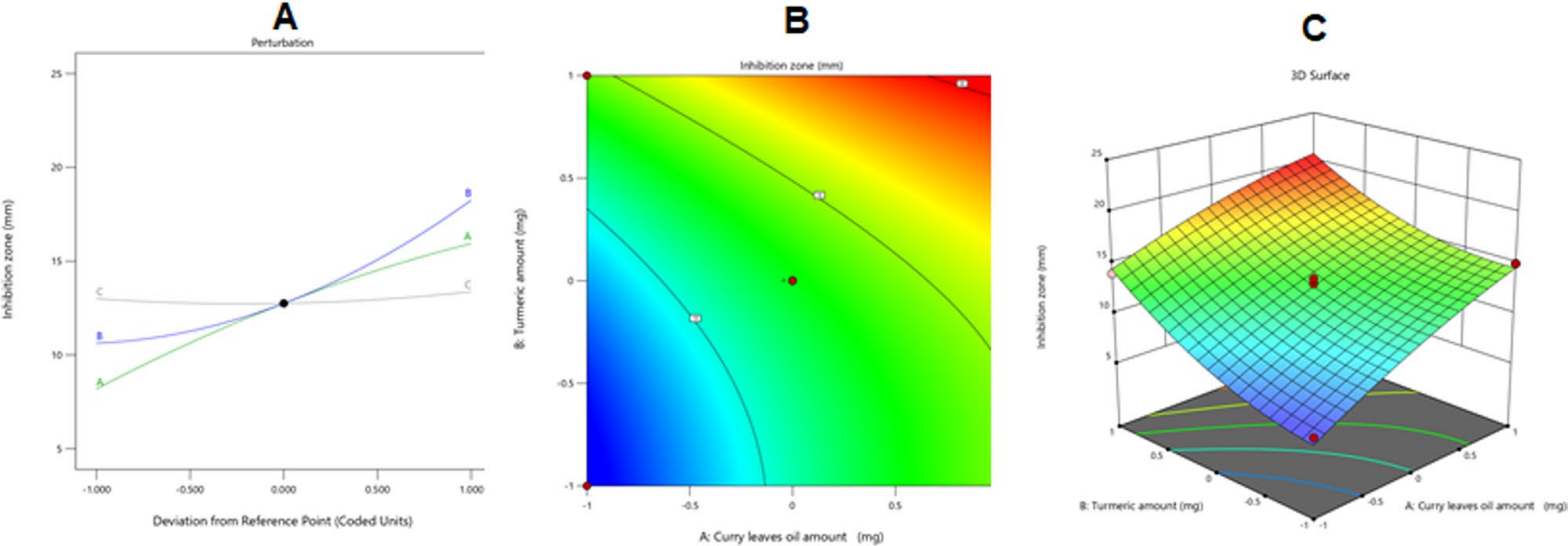
Perturbation (A), contour (B), and 3D surface (C) plots showing the effects of several independent parameters on the inhibition zones against *S. mutans* of various CrO-Tur-SNEDDS formulations.

Figure 7 further shows how the examined parameters affected the bacterial growth inhibition zones of the CrO-Tur-SNEDDS against *S.mutans* in its main effect, contour, and 3D surface graphs.

As would be expected from the preceding graph and equation, CrO and Tur had a significant capability for extending the growth inhibition zones of *S. mutans*, while Smix 2:1 did not have a crucial impact on the same response.

The antibacterial impact of CrO might be attributed to its content of terpenes, such as β-thujene, α-pinene, β-pinene, (E-)-β-ocimene, and δ-elemene. Some mechanisms have been suggested for the antibacterial effects of such components. For instance, disruption of the cell membrane and alterations to the ion channels (Na+, K+, Ca2+, or Cl) within the cell membrane may increase permeability and result in the release of essential intracellular components (Oz et al., 2015), as well as the inhibition of target enzymes (Ouattara et al., 1997). Recently discovered CrO carbazole alkaloids with pancreatic lipase inhibitory activity need to be further studied for their possible role in antimicrobial activity (Erkan et al., 2012).

Factor B (Tur amount) was found to have a considerable antmicrobial effect, which might be due to its curcumin content. Previous research has demonstrated that curcumin has a wide range of antibacterial activities at several pharmacological sites of action (Sharifi et al., 2020). It has been discovered that curcumin can modify gene expression and prevent bacterial DNA replication. Additionally, it impairs bacterial cell membrane integrity and decreases microorganism motility (Tyagi et al., 2015). Curcumin inhibits the polymerization of FtsZ protofilaments and interferes with GTPase activity in the cytoskeleton of *Bacillus subtilis, Escherichia coli* (Kaur et al., 2010), and *Staphylococcus aureus* (Teow et al., 2016), according to *in-vitro* studies.

Through the mechanism of binding to Toll-like receptors, curcumin can affect bacterial cell division and proliferation. Other research studies have determined that curcumin enhances an apoptosis-like response in *E. coli* (Yun & Lee, 2016). The antibiofilm properties of curcumin have also been reported for various bacterial species, including *P. aeruginosa, Proteus mirabilis, E. coli,* and *S. mutans* (Li et al., 2019).

### 3.4. Optimization of the CrO-Tur-SNEDDS

**Figure 8.**
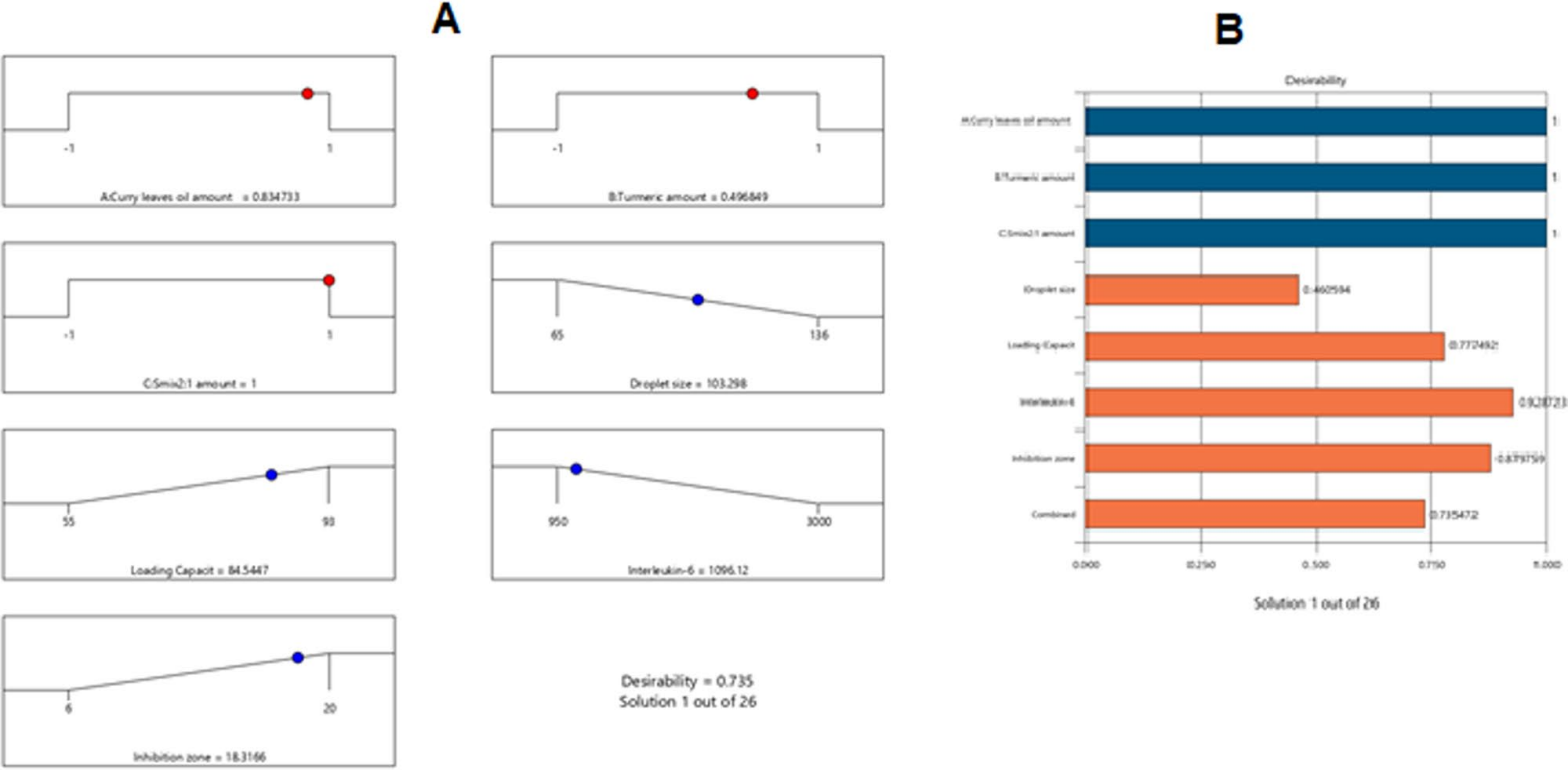
Bar chart with desirability ramp for process optimization. The levels of the parameters that were studied and the expected estimations for the dependent variables of the optimal formulation are shown in the desirability ramp in (A). (B) The bar graph shows the overall assessments of desirability.

The optimized nanoemulsion formulation was made using the most relevant attributes based on the obtained data. The optimal combination consisted of 240 mg of CrO, 42.5 mg of Tur, and 600 mg of Smix 2:1, according to the employed software, which also recommended several other alternatives that represented different combinations of the factors being studied. As previously stated, Tur was more soluble in surfactants with a low HLB than in those with a high HLB. However, since the HLB for a surfactant must be 10 or above to generate an oil/water (o/w) nanoemulsion (Azeem et al., 2009), a mixture of two surfactants was prepared and blended in the right proportions to create a surfactant mixture with an HLB of 10. So, a surfactant blend was developed by admixing Plurol and Labrasol in a ratio of 1:4.5 to prepare a mixture with an HLB value of 10. The developed optimized formulation had a Tur-loading capacity of 88%, a droplet size of 101 nm, a growth inhibition zone of 18 mm, and an IL-6 serum level of 1078 U/ml, and it acquired a desirability of 0.735. Figure 8 displays the desirability ramp, levels of the independent variables, and expected values of the measured responses for the best formulation.

Table 4 shows that the best formulation’s actual and predicted values were almost identical, with no discernible differences (*p* > .05), supporting the accuracy, validity, and precision of the equations.

**Table 4.** Values obtained experimentally and in reality for the ideal nanoemulsion formulation.

Solution	CrO (mg)	Tur (mg)	S_mix2:1_ (mg)	Droplet size (nm)	Tur loading capacity(%)	Il-6 level (U/ml)	Inhibition zone (mm)	desirability
Predicated value	240	42.5	600	101	88	1078	18	0.735
Experimental value	240	42.5	600	103.298	84.54	1096.12	18.31	0.735

### 3.5. Stability index determination

After the heat-cool test, no considerable differences in the optimal formulation’s uniformity and integrity were seen. When examining the stability of nanoemulsions, the stability index is of crucial relevance. The generated optimal nanoemulsion maintained an acceptable level of stability, as evidenced by its stability index value of 93% and the ability of the optimal levels defined by the design to create a high-quality, stable nanoemulsion (Choi & McClements, 2020). No phase separation was noted after the ideal sample had been centrifuged.

### 3.6. Characterization of the hyaluronic acid nanoemulgel

#### 3.6.1. In-vitro release of Tur from nanoemulgels

**Figure 9.**
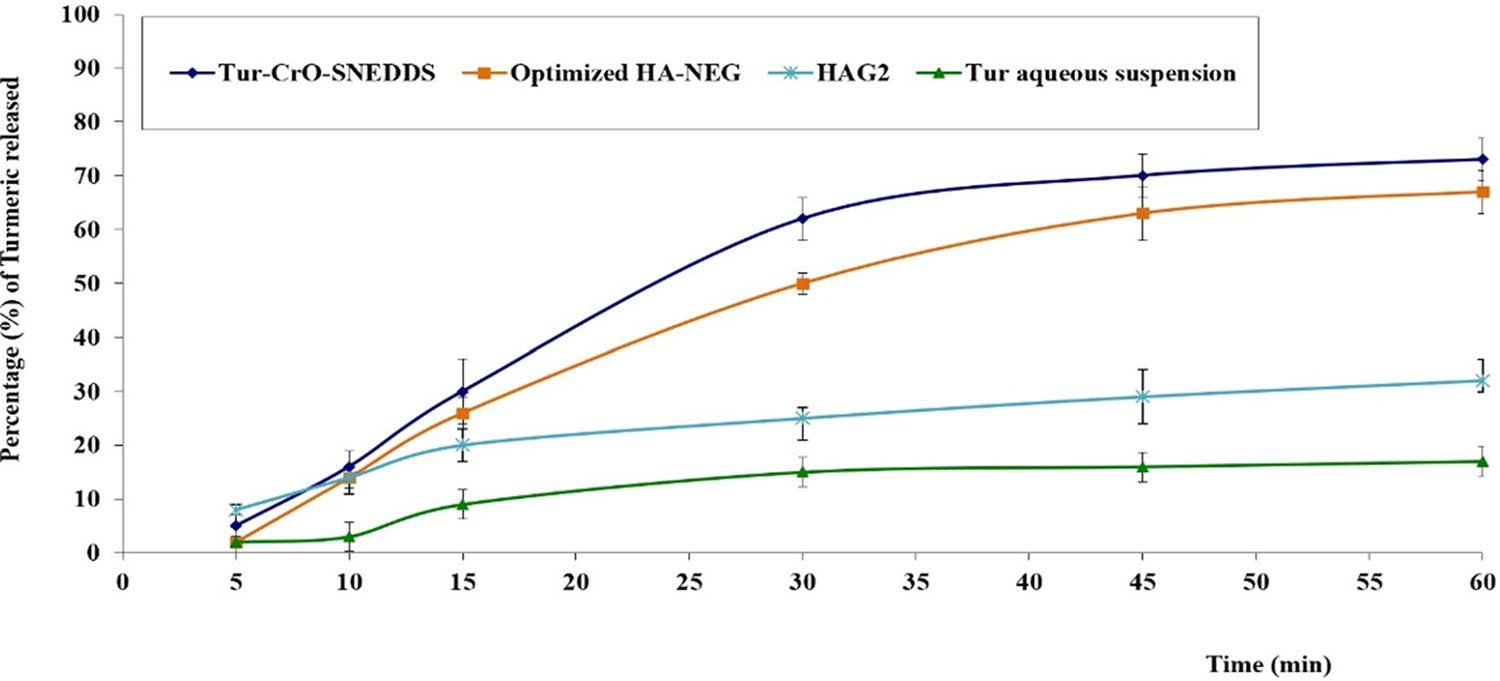
The *in-vitro* release behavior of Tur from the examined formulations N.B. HAG2 is the gel formulation that encompassed the Tur powder along with the CrO.

Figure 9 displays the drug release profiles for the formulations that were studied. A very low cumulative percentage of Tur was released after 60 minutes (17 + 2%), and this may have been related to the poor water solubility of Tur.

The percentage of release of Tur from the CrO-Tur-SNEDDS was 73 ± 3%. This may have been due to the nanoemulsion’s content of amphiphilic molecules, which may improve drug release by enhancing Tur dissolution, as well as the small size of the manufactured nanoglobules of the emulsion, which may have offered a broader surface area for drug release; they may have been responsible for the optimized formulation’s high release percentage compared with the percentages of the other formulations (Hosny et al., 2021).

Concurrently, the formulation composed of HA loaded with the optimal formulation had a drug release percentage of 67 ± 4%. That percentage was lower than the percentage from the dipsersion of the ideal formulation. This might have been due to the high viscosity of the HA nanoemulgel formulation, which may have constrained the drug release (Salem et al., 2022). The gel formulation that encompassed the Tur powder along with the CrO achieved a Tur release percentage of 32 ± 4%. Such a small amount of Tur released from this formulation could be associated with the absence of the nanodelivery system and the use of the drug and oil in their plain forms, which deprived this formulation of the dissolution-enhancing effect of the surfactants usually included in the nanoemulsion.

#### 3.6.2. Ex-vivo transmucosal permeation study

As can be seen from Table 5, formulation NG1, which was made up of the ideal CrO-Tur-SNEDDS loaded into the HA gel, had a Q_24_ value of 7530 ± 215 µg/cm^2^ and had a permeation 6.76 times higher than that of the Tur aqueous suspension, which had a Q_24_ of 1113 ± 55 µg/cm^2^. With this outcome, it became clear that nanoemulsions played a substantial role in improving Tur penetration through the mucosa. The presence of surfactants in the nanoemulsion had a crucial role in boosting drug permeation, since they may have helped drugs diffuse out of formulations and have a significant impact on fluidizing mucosal cell membranes, which increased Tur penetration (Salem et al., 2020). The same outcomes could be seen upon comparing the drug permeation from NG2, which was composed of HA gel containing Tur powder and CrO but not a SNEDDS and achieved a Q_24_ of only 2811 ± 91 µg/cm^2^. These findings also confirmed the considerable ability of nanoemulsions to enhance the permeation of biological membranes by active agents.

**Table 5.** *Ex-vivo* trans-mucosal permeation parameters of Tur from different tested gel formulations.

Parameters of permeation	NG1	NG2	NG3	NG4	Tur Aqueous suspension
Cumulative amount permeated Q_24_ (μg/cm^2^)^a^	7530 ± 215	2811 ± 91	4311 ± 128	5986 ± 101	1113 ± 55
Steady state flux, Jss^b^, (μg/cm^2^.min)	45.2	15.4	27.6	31.8	6.2
Permeability coefficient, P^c^, (cm/min)	8.1 × 10^-3^	3.1 × 10^-3^	5.2 × 10^-3^	6.6 × 10^-3^	1.8 × 10^-3^
Diffusion coefficient, D^d^, (cm^2^/min)	5.11 × 10^-4^	2.11 × 10^-4^	3.21 × 10^-4^	3.98 × 10^-4^	1.17 × 10^-4^
Enhancement factor (F_en_)^e^	6.76	2.52	3.87	5.378	–

N.B. NG1 is the optimal CrO-Tur-SNEDDS loaded into HA gel; NG2 is the HA gel containing the Tur as a powder and CrO distributed as an oil; NG3 is the HPC gel loaded with the optimized CrO-Tur-SNEDDS; and NG4 is the HA gel containing the nanoemulsion produced using oleic acid rather than CrO as a component of the SNEDDS.

^a^ Q is the cumulative Tur amount that permeated the membrane per unit area.

^b^ Jss was obtained from the slope of the curve plotted between the cumulative amount that permeated the membrane and the time.

^c^ P is the Tur Jss/original concentration.

^d^ D was calculated from the slope of the curve plotted between the cumulative amount that permeated the membrane and the square root of time.

^e^ EF is the cumulative amount that permeated Q of the studied formulations divided by the cumulative amount that permeated the Tur suspension.

Formulation NG3, which contained the optimal CrO-Tur-SNEDDS loaded into HPC, achieved a Q_24_ value of 4311 ± 128 µg/cm^2^; this was much lower than the value of formulation NG1, which was made up of the ideal CrO-Tur-SNEDDS loaded into HA gel and achieved drug permeation of up to 7530 ± 215 µg/cm^2^. These findings affirmed the enhanced ability of HA to permeate biological membranes. The outstanding permeation-boosting capacity of HA could be ascribed to the substance’s hygroscopic nature. It is a hygroscopic molecule that can hydrate the cell membrane and alter the microstructure of lipid self-assembly, ultimately making it easier for substances to pass through a cell wall (Yuan et al., 2022).

For formulation NG4, which contained oleic acid instead of CrO in the developed nanoemulsion, the permeation parameters were also much lower than those of NG1, which contained CrO among its components. For example, NG4 had a Q_24_ value of 5986 ± 101 µg/cm^2^ compared with the NG1 value of 7530 ± 215 µg/cm^2^. Such a reduction in drug permeation affirmed the importance of CrO in improving drug permeation. Its better action might have been due to its terpene content. The presence of terpene compounds increased the drug solubility in membrane lipids and the lipid/protein arrangement was disrupted during permeation enhancement (Sapra et al., 2008). Therefore, there seems to be a lot of potential for the usage of terpenes in topical and buccal formulations.

#### 3.6.3. Determination of zone of inhibition against S. mutans

The inhibition zones against *S. mutans* produced by the fabricated gel formulations varied. The growth inhibition zones were 19 ± 1 mm for NG1, 11 ± 0.5 mm for NG2, 18 ± 1.5 mm for NG3, 13 ± 1 mm for NG4, and 8 ± 0.5 mm for the Tur aqueous dispersion. The high value for the microbial growth inhibition zone for NG1 might have had various causes.

First of all, such remarkable antibacterial activity might have been due to the nanosize of NG1. The small size of the nanoemulsion’s droplets might have offered a greater surface area for contact with the bacterial cell membrane (Alhakamy et al., 2022). Additionally, the CrO and Tur content of the nanoemulsion contributed to the antibacterial action of NG1. As previously mentioned, it may be the presence of terpenes such β-thujene, α-pinene, β-pinene, (E-)-β-ocimene, and δ-elemene in the CrO that give it its antibacterial effects. There have been some proposed mechanisms for the antibacterial properties of such components. For example, cell membrane disruption and changes to the ion channels (Na+, K+, Ca2+, or Cl) within the cell membrane may enhance permeability and cause the release of vital intracellular components, as well as inhibit the target enzymes (Erkan et al., 2012; Oz et al., 2015). Furthermore, curcumin, the main ingredient in Tur, has a variety of antibacterial effects that can be mediated by many mechanisms, according to a prior study (Sharifi et al., 2020). Curcumin has been shown to alter gene expression and inhibit bacterial DNA replication. Additionally, it might reduce the motility of microorganisms and damage the integrity of bacterial cell membranes (Tyagi et al., 2015).

Formulation NG2 attained an inhibition zone of 11 ± 0.5 cm. Such a sharp decrease compared to optimized formulation might be explained by the lack of the nanosized drug delivery system and the use of CrO as an oil and Tur as a powder. This outcome confirmed the pivotal role of the nanosized delivery systems in conveying drugs to required sites.

Formulation NG3 achieved a growth inhibition zone of 18 ± 1.5 cm, a result very close to the value for formulation NG1. The only difference between NG1 and NG3 was the type of polymer used in developing the gel base: HA was the gel base in NG1 and HPC was the gel base in NG3. The result showed that the type of polymer used in producing the gel did not have a significant impact on the inhibition zone. Formulation NG4 achieved an inhibition zone of 13 ± 1 cm. This low value might have been due to the use of oleic acid instead of CrO in this formulation; this would have caused the formulation to lose the antibacterial action of CrO. Eventually, the Tur aqueous suspension had the smallest inhibition zone (i.e. 8 ± 0.5 cm). This antibacterial activity was mainly due to the curcumin content of Tur, and the low value obtained proved the importance of combining Tur and CrO in the developed formulation to boost its antibacterial activity.

#### 3.6.4. Determination of IL-6 serum levels

Group 1, the animals that received the optimal formulation loaded in an HA base, attained an IL-6 serum value of 900 ± 40 U/ml, which was the lowest value of all tested groups. This affirmed the significant anti-inflammatory effect of the HA gel containing the optimal nanoemulsion. These results could be ascribed to the combined anti-inflammatory effect of Tur and CrO. Curcumin, the main component of Tur, exerts anti-inflammatory effects through regulating inflammatory signaling pathways and lowering the production of inflammatory mediators, as previously stated in the current work (Medzhitov, 2008; Zhang et al., 2019). The presence of terpene alkaloids in CrO may also play a role in reducing the synthesis and activity of prostaglandins, proteases, and lysosomes, and hence enhances the oil’s anti-inflammatory effect (Gupta et al., 2010).

HA also contributed to the anti-inflammatory effect of the formulation used in the treatment of Group1. HA serves a number of extracellular roles that are essential for preserving healthy gum tissue, including acting as a barrier to plaque bacteria (Jain, 2013). HA was employed in one study (Chen et al., 2019) to treat human gingival fibroblast wounds and inflammation brought on by *Porphyromonas gingivalis* (hepatocyte growth factors [HGFs]). The findings showed that HA reduced the levels of IL-1, IL-6, IL-8, IL-4, and IL-10. Additionally, HA prevented *P. gingivalis*‒induced ERK and p38 MAPK activation, as well as the expression of NF-кB, degradation of the nuclear factor kappa light polypeptide gene enhancer in the B-cell inhibitor, alpha (IкB), and activation of NF-кB. This demonstrated that HA could have favorable effects on oral wounds, gingivitis, and periodontal inflammation.

The preceding results were further confirmed when they were compared with those obtained upon treating the second group of animals with the optimal nanoemulsion formulation loaded into HPC instead of HA. The IL-6 serum level detected in the animals of Group 2 was 1175 ± 66 U/ml. This increase in Group 2 compared with Group 1 might have been due to the absence of HA in the formulation received by this group.

Group 3, which received topical HA gel loaded with the optimum formulation without Tur, acquired an IL-6 serum level of 1656 ± 93 U/ml. As could be noticed, there was an increase in the IL-6 level over Groups 1 and 2, and this signified the crucial anti-inflammatory impact of Tur. Group 4, which was treated with the HA gel base loaded with the optimum nanoemulsion with oleic acid instead of CrO, had an IL-6 serum level of 2389 ± 101 U/ml. Such an increment in the inflammatory mediator in Group 4 might be ascribed to the absence of CrO and its components, which have unique anti-inflammatory effects, as previously stated in the current article.

Group 5 was treated with the aqueous dispersion of Tur and achieved an IL-6 serum level of 2634 ± 117 U/ml. This high value of the cytokine IL-6 compared with the preceding groups affirmed the importance of combining Tur with CrO, in addition to the great impact of the nanosized drug delivery systems and the gel base HA. Finally, Group 6, which received normal saline, had an IL-6 value of 3000 ± 145 U/ml. This finding clarified the important anti-inflammatory action of the chosen active agents and fabricated formulations.

## 4. Conclusions

A successful and functional nanoemulsion was created by incorporating CrO and Tur into it. To develop the required drug delivery system, the optimal level of Smix was calculated using a pseudoternary phase diagram. The generated nanoemulsions, which had globules ranging from 72 ± 0.19 to 136 ± 2.2 nm in diameter and an appropriate homogeneous distribution, suggested that the systems were reasonably stable. The developed formulations achieved a medication-loading efficiency ranging from 55 ± 1.5 to 93 ± 2.1%, a bacterial growth inhibition zone of up to 20 ± 0.5 mm, and an IL-6 serum level of between 950 ± 10 and 3000 ± 25 U/ml. The optimum formulation was created using the response surface Box-Behnken design and contained 240 mg of CrO, 42.5 mg of Tur, and 600 mg of Smix 2:1. The best formulation was subsequently incorporated into an HA oral gel, where it displayed improved transmucosal permeability and sustained Tur release. When compared with the other evaluated formulations, the gel containing the optimized CrO-Tur-SNEDDS had the largest bacterial growth inhibition zone and the lowest IL-6 serum level in rats. Overall, this work demonstrated the potential of CrO-Tur‒based nanoemulsions loaded into an HA gel for providing the effective prevention of gingivitis.

## References

[CIT0001] Alexander A, Khichariya A, Gupta S, et al. (2013). Recent expansions in an emergent novel drug delivery technology: Emulgel. J Control Release 171:1–15. 23831051 10.1016/j.jconrel.2013.06.030

[CIT0002] Alhakamy NA, Hosny KM, Aldryhim AY, et al. (2022). Development and optimization of ofloxacin as solid lipid nanoparticles for enhancement of its ocular activity. J Drug Deliv Sci and Technol 72:103373.

[CIT0003] Ammon HP, Safayhi H, Mack T, Sabieraj J. (1993). Mechanism of anti‑inflammatory actions of curcumin and boswellic acids. J Ethnopharmacol 38:113–95. 8510458 10.1016/0378-8741(93)90005-p

[CIT0004] Ashrafizadeh M, Rafiei H, Mohammadinejad R, et al. (2020). Potential therapeutic effects of curcumin mediated by JAK/STAT signaling pathway: a review. Phytother Res 34:1745–60. 32157749 10.1002/ptr.6642

[CIT0005] Azeem A, Rizwan M, Ahmad FJ, et al. (2009). Nanoemulsion components screening and selection: a technical note. AAPS PharmSciTech 10:69–76. 19148761 10.1208/s12249-008-9178-x PMC2663668

[CIT0006] Baiju RM, Peter E, Varghese NO, Sivaram R. (2017). Oral health and quality of life: current concepts. J Clin Diagn Res 11:ZE21–ZE26. 28764312 10.7860/JCDR/2017/25866.10110 PMC5535498

[CIT0007] Blicher B, Joshipura K, Eke P. (2005). Validation of self-reported periodontal disease: a systematic review. J Dent Res 84:881–90. 16183785 10.1177/154405910508401003

[CIT0008] Califano JV. (2003). Position paper: periodontal diseases of children and adolescents. J Periodontol 74:1696–704. 7. 14682670 10.1902/jop.2003.74.11.1696

[CIT0009] Chainani-Wu N. (2003). Safety and anti‑inflammatory activity of curcumin: a component of turmeric (Curcuma longa). J Altern Complement Med 9:161–8. 12676044 10.1089/107555303321223035

[CIT0010] Chaturvedi TP. (2009). Uses of turmeric in dentistry: an update. Indian J Dent Res 20:107–9. 19336870 10.4103/0970-9290.49065

[CIT0011] Chen M, Li L, Wang Z, et al. (2019). High molecular weight hyaluronic acid regulates P. gingivalis–induced inflammation and migration in human gingival fibroblasts via MAPK and NF-kB signaling pathway. Arch Oral Biol 98:75–80. 30465936 10.1016/j.archoralbio.2018.10.027

[CIT0012] Choi SJ, McClements DJ. (2020). Nanoemulsions as delivery systems for lipophilic nutraceuticals: strategies for improving their formulation, stability, functionality and bioavailability. Food Sci Biotechnol 29:149–68. 32064124 10.1007/s10068-019-00731-4 PMC6992823

[CIT0013] Çıkrıkçı S, Mozioglu E, Yılmaz H. (2008). Biological activity of curcuminoids isolated from Curcuma longa. Rec Nat Prod 2:19–24.

[CIT0014] Date AA, Desai N, Dixit R, Nagarsenker M. (2010). Self-nanoemulsifying drug delivery systems: formulation insights, applications and advances. Nanomedicine 5:1595–616. 21143036 10.2217/nnm.10.126

[CIT0015] Erkan N, Tao Z, Rupasinghe HPV, et al. (2012). Antibacterial activities of essential oils extracted from leaves of Murraya koenigii by solvent-free microwave extraction and hydro-distillation. Nat Prod Commun 7:121–4. 22428264

[CIT0016] Friedman PM, Mafong EA, Kauvar AN, Geronemus RG. (2002). Safety data of injectable nonanimal stabilized hyaluronic acid gel for soft tissue augmentation. Dermatol Surg 28:491–4. 12081677 10.1046/j.1524-4725.2002.01251.x

[CIT0017] Gao Y, Zhuang Z, Lu Y, et al. (2019). Curcumin mitigates neuro-inflammation by modulating microglia polarization through inhibiting TLR4 axis signaling pathway following experimental subarachnoid hemorrhage. Front Neurosci 13:1223. 31803007 10.3389/fnins.2019.01223 PMC6872970

[CIT0018] Gift HC, Atchinson KA. (1995). Oral health, health and health related quality of life. Medical Care 33:557–77. 10.1097/00005650-199511001-00008 7475433

[CIT0019] Goa KL, Benfield P. (1994). Hyaluronic acid. Drugs 47:536–66. 7514978 10.2165/00003495-199447030-00009

[CIT0020] Gupta S, George M, Singhal M, et al. (2010). Leaves extract of murraya koenigii linn for anti-inflammatory and analgesic activity in animal models. J Adv Pharm Technol Res 1:68–77. Jan 22247833 PMC3255384

[CIT0021] Hosny KM, Alhakamy NA, Sindi AM, et al. (2020). Coconut oil nanoemulsion loaded with a statin hypolipidemic drug for management of burns: formulation and in vivo evaluation. Pharmaceutics 12:1061. 33171816 10.3390/pharmaceutics12111061 PMC7695003

[CIT0022] Hosny KM, Khallaf RA, Asfour HZ, et al. (2021). Development and optimization of cinnamon oil nanoemulgel for enhancement of solubility and evaluation of antibacterial, antifungal and analgesic effects against oral microbiota. Pharmaceutics 13:1008. 34371700 10.3390/pharmaceutics13071008 PMC8309164

[CIT0023] Hosny KM, Sindi AM, Alkhalidi HM, et al. (2021). Development of omega-3 loxoprofen-loaded nanoemulsion to limit the side effect associated with NSAIDs in treatment of tooth pain. Drug Deliv 28:741–51. 33840320 10.1080/10717544.2021.1909179 PMC8057080

[CIT0024] Hosny K, Asfour H, Rizg W, et al. (2021). Formulation, optimization, and evaluation of oregano oil nanoemulsions for the treatment of infections due to oral microbiota. Int J Nanomedicine 16:5465–78.,. 34413644 10.2147/IJN.S325625 PMC8370598

[CIT0025] Jain Y. (2013). Clinical evaluation of 0.2% hyaluronic acid containing gel in the treatment of gingivitis. Med J Dr. DY Patil Univ 6:416–20.

[CIT0026] Kahkhaie KR, Mirhosseini A, Aliabadi A, et al. (2019). Curcumin: a modulator of inflammatory signaling pathways in the immune system. Inflammopharmacology 27:885–900. 31140036 10.1007/s10787-019-00607-3

[CIT0027] Kaur S, Modi NH, Panda D, Roy N. (2010). Probing the binding site of curcumin in Escherichia coli and Bacillus subtilis FtsZ—a structural insight to unveil antibacterial activity of curcumin. Eur J Med Chem 45:4209–14. 20615583 10.1016/j.ejmech.2010.06.015

[CIT0028] Li Q, Sun J, Mohammadtursun N, et al. (2019). Curcumin inhibits cigarette smoke-induced inflammation via modulating the PPARgamma-NF-kappaB signaling pathway. Food Funct 10:7983–94. 31773117 10.1039/c9fo02159k

[CIT0029] Li X, Yin L, Ramage G, et al. (2019). Assessing the impact of curcumin on dual-species biofilms formed by Streptococcus mutans and Candida albicans. Microbiol Open 8:e937. 10.1002/mbo3.937 PMC6925172 31560838

[CIT0030] Mahmoud EA, Bendas ER, Mohamed MI. (2010). Effect of formulation parameters on the preparation of superporous hydrogel self-nanoemulsifying drug delivery system (SNEDDS) of carvedilol. AAPS PharmSciTech 11:221–5. 20127427 10.1208/s12249-009-9359-2 PMC2850453

[CIT0031] Math MV, Balasubramaniam P. (2003). Curry leaves (Murraya koenigii Spreng) and halitosis. Br Med J 19:211.

[CIT0032] Medzhitov R, (2008). Origin and physiological roles of inflammation. Nature 4:428–35. 10.1038/nature07201 18650913

[CIT0033] Motterlini R, Foresti R, Bassi R, Green CJ. (2000). Curcumin, an antioxidant and anti‑inflammatory agent, induces heme oxygenase‑1 and protects endothelial cells against oxidative stress. Free Radic Biol Med 28:1303–12. 10889462 10.1016/s0891-5849(00)00294-x

[CIT0034] Mudge E, Chan M, Venkataraman S, et al. (2016). Curcuminoids in turmeric roots and supplements: method optimization and validation. Food Anal Methods 9:1428–35.

[CIT0035] Nguyen HA, Anderson CA, Miracle CM, Rifkin DE. (2017). The association between depression, perceived health status, and quality of life among individuals with chronic kidney disease: an analysis of the national health and nutrition examination survey 2011–2012. Nephron 136(2):127–35. 10.1159/000455750 28249290

[CIT0036] Nithya RJ, Gala VC, Chaaya SS. (2013). Inhibitory effects of plant extracts on multi-species dental biofilm formation-in vitro. Int J Pharma Bio Sci 4:487–95.

[CIT0037] Ouattara B, Simard RE, Holley RA, et al. (1997). Antibacterial activity of selected fatty acids and essential oils against six meat spoilage organisms. Int J Food Microbiol 37:155–62. 9310850 10.1016/s0168-1605(97)00070-6

[CIT0038] Oz M, Lozon Y, Sultan A, et al. (2015). Effects of monoterpenes on ion channels of excitable cells. Pharmacol Ther 152:83–97. 25956464 10.1016/j.pharmthera.2015.05.006

[CIT0039] Patel VF, Liu F, Brown MB. (2011). Advances in oral transmucosal drug delivery. J Control Release 153:106–16. 21300115 10.1016/j.jconrel.2011.01.027

[CIT0040] Pavoni L, Perinelli DR, Bonacucina G, et al. (2020). An overview of micro- and nanoemulsions as vehicles for essential oils: formulation, preparation and stability. Nanomaterials (Basel) 10:135. 31940900 10.3390/nano10010135 PMC7023169

[CIT0041] Pinheiro LC, Tan X, Olshan AF, et al. (2017). Examining health-related quality of life patterns in women with breast cancer. Qual Life Res 26:1733–43. 10.1007/s11136-017-1533-5 PMC5539913 28247314

[CIT0042] Rahimifard M, Maqbool F, Moeini-Nodeh S, et al. (2017). Targeting the TLR4 signaling pathway by polyphenols: a novel therapeutic strategy for neuroinflammation. Ageing Res Rev 36:11–9. 28235660 10.1016/j.arr.2017.02.004

[CIT0043] Rizg WY, Hosny KM, Elgebaly SS, et al. (2021). Preparation and optimization of garlic oil/apple cider vinegar nanoemulsion loaded with minoxidil to treat alopecia. Pharmaceutics 13:2150. 34959435 10.3390/pharmaceutics13122150 PMC8706394

[CIT0044] Salem HF, El-Menshawe SF, Khallaf RA, et al. (2020). A novel transdermal nanoethosomal gel of lercanidipine HCl for treatment of hypertension: optimization using Box-Benkhen design, in vitro and in vivo characterization. Drug Deliv Transl Res 10:227–40. 31625026 10.1007/s13346-019-00676-5

[CIT0045] Salem HF, Nafady MM, Ewees MGE, et al. (2022). Rosuvastatin calcium-based novel nanocubic vesicles capped with silver nanoparticles-loaded hydrogel for wound healing management: optimization employing Box-Behnken design: in vitro and in vivo assessment. J Liposome Res 32:45–61. 33353435 10.1080/08982104.2020.1867166

[CIT0046] Sapra B, Jain S, Tiwary AK. (2008). Percutaneous permeation enhancement by terpenes: mechanistic view. Aaps J 10:120–32. 18446512 10.1208/s12248-008-9012-0 PMC2751457

[CIT0047] Schwartzmann L. (2003). Quality of life related to health: conceptual aspects. Science Nurs 2:9–2.

[CIT0048] Sharifi S, Fathi N, Memar MY, et al. (2020). Anti-microbial activity of curcumin nanoformulations: new trends and future perspectives. Phytother Res 34:1926–46. 32166813 10.1002/ptr.6658

[CIT0049] Sravani K, Suchetha A, Mundinamane DB, et al. (2015). Plant products in dental and periodontal disease: an overview. Int J Med Dent Sci 4:913–21.

[CIT0050] Sun H, Liu K, Liu W, et al. (2012). Development and characterization of a novel nanoemulsion drug-delivery system for potential application in oral delivery of protein drugs. Int J Nanomedicine 7:5529–43. 23118537 10.2147/IJN.S36071 PMC3484902

[CIT0051] Tanaka T, Narazaki M, Kishimoto T. (2014). IL-6 in inflammation, immunity, and disease. Cold Spring Harb Perspect Biol 6:a016295. 25190079 10.1101/cshperspect.a016295 PMC4176007

[CIT0052] Teow SY, Liew K, Ali SA, et al. (2016). Antibacterial action of curcumin against Staphylococcus aureus: a brief review. J Trop Med 2016:2853045. 27956904 10.1155/2016/2853045 PMC5124450

[CIT0053] Toda S, Miyase T, Arich H. (1985). Natural antioxidants. Antioxidative compounds isolated from rhizome of Curcuma longa L. Chem Pharmacol Bul 33:1725–8. 10.1248/cpb.33.1725 4042250

[CIT0054] Tyagi P, Singh M, Kumari H, et al. (2015). Bactericidal activity of curcumin is associated with damaging of bacterial membrane. PLoS ONE 10:e0121313. 25811596 10.1371/journal.pone.0121313 PMC4374920

[CIT0055] Van der Velden U. (2006). The significance of supragingival plaque accumulation in periodontal disease. Int J Dent Hyg 4:11–4. 16965528 10.1111/j.1601-5037.2006.00196.x

[CIT0056] WHOQoL Group. (1995). The World Health Organization quality of life assessment (WHOQoL): position paper from the World Health Organization. Soc Sci Med 41:1403–9. 8560308 10.1016/0277-9536(95)00112-k

[CIT0057] Xiang J, Fang X, Li X. (2002). Transbuccal delivery of 2’,3’-dideoxycytidine: in vitro permeation study and histological investigation. Int J Pharm 231:57–66. 11719014 10.1016/s0378-5173(01)00865-1

[CIT0058] Yuan M, Niu J, Xiao Q, et al. (2022). Hyaluronan-modified transfersomes based hydrogel for enhanced transdermal delivery of indomethacin. Drug Deliv 29:1232–42. 35403516 10.1080/10717544.2022.2053761 PMC9004534

[CIT0059] Yun DG, Lee DG. (2016). Antibacterial activity of curcumin via apoptosis-like response in Escherichia coli. Appl Microbiol Biotechnol 100:5505–14. 26960318 10.1007/s00253-016-7415-x

[CIT0060] Zhang J, Zheng Y, Luo Y, et al. (2019). Curcumin inhibits LPS-induced neuroinflammation by promoting microglial M2 polarization via TREM2/ TLR4/ NF-kappaB pathways in BV2 cells. Mol Immunol 116:29–37. 31590042 10.1016/j.molimm.2019.09.020

[CIT0061] Zhu T, Chen Z, Chen G, et al. (2019). Curcumin attenuates asthmatic airway inflammation and mucus hypersecretion involving a PPARgamma-dependent NF-kappaB signaling pathway in vivo and in vitro. Mediators Inflamm 2019:4927430. 31073274 10.1155/2019/4927430 PMC6470457

